# miR-18a Mediates Immune Evasion in ER-Positive Breast Cancer through Wnt Signaling

**DOI:** 10.3390/cells11101672

**Published:** 2022-05-18

**Authors:** Madhumathy G. Nair, Apoorva D, Chandrakala M, Snijesh VP, Sharada Patil, Anupama CE, Geetashree Mukherjee, Rekha V. Kumar, Jyothi S. Prabhu, Sridhar TS

**Affiliations:** 1 Division of Molecular Medicine, St. John’s Research Institute, St. John’s Medical College, Bangalore 560034, India; apoorva.md@sjri.res.in (A.D.); chandrakala.m@sjri.res.in (C.M.); snijesh.vp@sjri.res.in (S.V.); sharada.sharan@gmail.com (S.P.); anupamace@sjri.res.in (A.C.); jyothi@sjri.res.in (J.S.P.); 2 Kidwai Memorial Institute of Oncology, Bangalore 560029, India; mukherjeegeeta@yahoo.co.in (G.M.); rekha_v_kumar@yahoo.co.in (R.V.K.); 3 Histopathology, Tata Medical Center, Kolkata 700160, India; 4 Histopathology, Sri Shankara Cancer Hospital and Research Centre, Bengaluru 560004, India; 5 Department of Biological Sciences, Indian Institute of Science Education and Research, Berhampur 760010, India; sridhar@iiserbpr.ac.in

**Keywords:** miR-18a, ER-positive breast cancer, Wnt pathway, immune modulation, immunotherapy

## Abstract

ER-positive (ER+) breast cancer is considered immunologically ‘silent’ with fewer tumor-infiltrating immune cells. We have previously demonstrated the role of miR-18a in mediating invasion and poor prognosis in ER+ breast cancer by activation of the Wnt signaling pathway. Here, we explored the immune-modulatory functions of high levels of miR-18a in these tumors. A microarray-based gene expression analysis performed in miR-18a over-expressed ER+ breast cancer cell lines demonstrated dysregulation and suppression of immune-related pathways. Stratification of the ER+ tumor samples by miR-18a levels in the TCGA and METABRIC cohort and immune cell identification performed using CIBERSORT and Immune CellAI algorithms revealed a higher proportion of T-regulatory cells (*p* < 0.001) and a higher CD4/CD8 ratio (*p* < 0.01). miR-18a over-expressed MCF7 co-cultured with THP-1 showed decreased antigen presentation abilities and increased invasiveness and survival. They also promoted the differentiation of pro-tumorigenic M2 macrophages. Inhibition of the Wnt pathway in miR-18a over-expressed cells brought about the restoration of TAP-1, a protein critical for antigen presentation. Examination of tumor specimens from our case series showed that miR-18a high ER+ tumors had a dense lymphocyte infiltrate when compared to miR-18a low tumors but expressed a higher CD4/CD8 ratio and the M2 macrophage marker CD206, along with the invasive marker MMP9. We report for the first time an association between miR-18a-mediated Wnt signaling and stromal immune modulation in ER+ tumors. Our results highlight the possibility of formulating specific Wnt pathway inhibitors that may be used in combination with immune checkpoint blockers (ICB) for sensitizing ‘immune-cold’ ER+ tumors to immunotherapy.

## 1. Introduction

Hormone-receptor-positive breast cancer accounts for nearly 70% of all breast cancers. This subtype of breast cancer has a relatively good prognosis when compared to the triple-negative breast cancer (TNBC) subtype but is often featured by late relapse [1]. ER+ metastatic breast cancer (mBC) is often aggressive, and the median five-year survival rate is 27%, which is an indication of an urgent need for developing improved treatment strategies for this group of patients [2]. Resistance to endocrine therapy in ER+ breast cancer patients may be attributed to numerous mechanisms, including diminished ERα signaling that may be silenced by multiple factors such as microRNA-mediated gene silencing [3]. microRNAs are highly conserved, small regulatory, non-coding RNA molecules that regulate post-transcriptional gene silencing by mRNA cleavage or translation repression. Deregulated expression of miRNAs is implicated in the clinical progression of cancer, leading to therapy resistance, invasion, and metastasis [4]. We have previously shown that miR-18a, which is known to directly repress ERα translation and block the protective effects of estrogen, activates the Wnt pathway, alters the cytoskeletal organization, and imparts the migratory ability to ER+ breast cancer cells. High levels of miR-18a also serve as a poor prognostic marker in ER+ breast tumors [5].

Immune checkpoint blockade (ICB) therapy has been successful in demonstrating clinical responses only in TNBC that is associated with dense tumor-infiltrating lymphocytes (TILs). ER+ breast cancer is generally deemed immunologically ‘cold’ with lesser immune infiltrate and minimal response to ICB. Currently, there is no FDA-approved ICB therapy for ER+ breast cancer, owing to its low mutational burden, low PD-L1 expression, and low TILs [6,7]. Hence, it is critical to identify signaling pathways and thereby therapeutic targets that can be used in combination with ICB to augment the anti-tumor immune response and sensitize these tumors to immunotherapy. In this study, we report a novel mechanism by which immune suppression is mediated by high levels of miR-18a in ER+ breast cancer through activation of the Wnt pathway.

## 2. Materials and Methods:

### 2.1. Cell Lines, Culture, and Transfection with miR-18a Mimics

The breast cancer cell line MCF7 was obtained from the American Type Culture Collection (ATCC), Manassas, VA. The culture conditions and the phenotypic characterization of the cell line have been reported previously [8]. ZR-75-1 and THP-1; a monocytic cell line was obtained from NCCS (Pune, India; where cell authentication was performed using STR profiling) and cultured in RPMI supplemented with 10% FBS. Transfection with micrON™ mimic for miR-18a-5p was performed as reported previously [5]. A non-specific miRNA mimic was used as the scrambled or negative control. The final dose of the mimic and scrambled was 50 nM. The cells over-expressing miR-18a will be referred to hereafter as MCF7-miR-18a-mimic or ZR-75-1-miR-18a-mimic, and the cells transfected with the scrambled control will be referred to as MCF7-negative control or ZR-75-1-negative control. The transfection efficiency was evaluated by assessing the levels of miR-18a by q-PCR and levels of the miR-18a targets by blot after 48 or 72 h as reported previously [5]. If not specified otherwise, miR-18a refers to miR-18a-5p in this study.

### 2.2. Microarray Analysis

Microarray-based global gene expression analysis of hsa-miR-18a-5p-over-expressing MCF7 cells was performed using 8 × 60 v3 Agilent arrays (Genotypic Technology, Bangalore) as described previously [5]. The microarray data analysis was carried out using r package *limma* (v3.42.2) [9]. Significant differentially expressed genes (DEGs) between miR-18a high and miR-18a low groups were filtered based on absolute fold change (FC) ≥ 2 and adjusted *p* ≤ 0.05. The heatmap and volcano plots representing DEGs were created using r packages such as *pheatmap* (v1.0.12) [10] and *EnhancedVolcano* (v 1.4.0) respectively. Gene ontology and pathway analysis of DEGs were performed using the ToppGene suite [11].

### 2.3. Quantitative Real Time PCR

Quantitation of RNA, cDNA synthesis, and q-PCR experiments were performed as reported previously [12,13]. Primers for all genes were designed using the software Primer3Plus and manufactured by Eurofins, Bangalore, India. The primer sequences for the genes tested are given in Supplementary Table S1.

### 2.4. Immune Cell Analysis

The ER+ HER2- tumors of the TCGA-PanCancer Atlas (*n* = 479) and the METABRIC-Nature 2012 and Nat Commun 2016 cohorts (*n* = 883) were segregated based on the upper and lower quartiles of miR-18a expression. The TCGA data were accessed from the TCGA Research Network: https://www.cancer.gov/tcga (accessed on 15 November 2020), and the METABRIC data were accessed from the European Genome-phenome Archive [14]. The normalized gene expression data with standard annotation files from the TCGA and the METABRIC cohorts were used for deconvolution of infiltrating immune populations by CIBERSORT. The CIBERSORT deconvolution approach helps to explore the cellular composition of complex tissues that can be estimated based on standardized gene expression data, which indicates the abundance of specific immune cell types. CIBERSORT was run with the following options: relative and absolute modes together, LM22 signature gene file, 1000 permutations, and quantile normalization disabled. Using the filtered data, the proportions of immune cells in the miR-18a high- and low-expressing breast tumor samples were displayed in the form of a proportion plot. The normalized gene expression data with standard annotation files from the TCGA cohort were also uploaded to the Immune Cell Abundance Identifier (ImmuCellAI), which uses a gene set signature-based method to precisely estimate the infiltration score of 24 immune cell types, including 18 T-cell subsets [15].

### 2.5. THP-1 Macrophage Differentiation Assay

THP-1 cells were differentiated to M0 macrophages by treatment with 150 nM of phorbol-12-myristate 13-acetate (PMA) for 24 h. The cells were then provided with fresh 10% RPMI medium without PMA for a day to allow cell recovery. The differentiated M0 macrophages were further induced to the M1 phenotype by treatment with 15 ng/mL lipopolysaccharide (LPS) and to the M2 phenotype by treatment with 25 ng/mL of both IL-4 and IL-13, respectively, for 48 h. The cell differentiation was verified by evaluating cell adhesion and spreading under an optical microscope.

### 2.6. Immunophenotyping of Monocytes

THP-1 cells were differentiated to M0 macrophages by PMA induction as described above. These cells were trypsinized and counted to 1 × 10^5^ cells/mL. THP-1 cells that were not activated with PMA (untreated) were also used for analysis. Cells were washed with PBS, fixed in 10% PFA for 10 min, followed by permeabilization in 0.2% Triton X-100 (Calbiochem) in PBS for 10 min. Cells were then blocked with 0.5% filter-sterilized BSA for 25 min and then incubated for 1 h in primary antibody for CD14 (61D3) Mouse mAb (FITC conjugate) at a dilution of 1:25. Cells were washed and resuspended in 500 μL of PBS and analyzed using a FACSCalibur cytometer (BD Biosciences). Unstained cells were used as a control to determine the regions that demarcate low (M0 macrophages) and high (monocytes) FITC-labeled populations of cells. The FL1-H channel was used to detect CD14, and an FSC-H vs. FL1-H plot was used to analyze the percentage of CD14+ cells.

### 2.7. Trans-Well Co-Culture Assay

MCF7 and THP-1 cells’ co-culture system was performed using 0.4 μm cell culture inserts in a 12-well plate that allows the diffusion of media components while preventing cell migration/phagocytosis. MCF7 cells were seeded in 12-well plates and transfected with miR-18a mimic or the scrambled miRNA as described above. After 72 h, THP-1 cells were seeded in trans-well inserts and placed with MCF7-miR-18a-mimic or MCF7-negative control. Both THP-1 and MCF7-miR-18a-mimic or MCF7-negative control cells were harvested after 4 days to assay for THP-1 differentiation and to assay for changes to MCF7-miR-18a-mimic or MCF7-negative control cells.

Further, to assay for M1/M2 phenotypic changes induced by MCF7-miR-18a-mimic or MCF7-negative control cells, THP1 cells were differentiated in trans-well inserts with 150 nM of PMA for 24 h and placed with MCF7-miR-18a-mimic or MCF7-negative control cells. Both differentiated THP-1 and MCF7-miR-18a-mimic or MCF7-negative control cells were harvested after 2 days to assay for M1/M2 macrophage markers and to assay the effect on MCF7-miR-18a-mimic or MCF7-negative control cells.

### 2.8. Western Blot

The protein expression was assayed as reported previously [13]. The list of antibodies used is listed in the Supplementary Materials (Supplementary Table S2). Densitometric analysis was performed using quantity one software (Bio-Rad) as reported previously [13].

### 2.9. C59-Wnt Pathway Inhibition

C59, a potent Wnt signaling pathway inhibitor, was used for impeding this pathway. MCF7 cells were seeded in 12-well plates and transfected with MCF7-miR-18a-mimic or scrambled control as described above. After 4 h of transfection, 10 µM C59/solvent control was added to MCF7-miR-18a-mimic. After 72 h, THP-1 cells were seeded in trans-well inserts and placed with MCF7-miR-18a-mimic or MCF7-negative control. MCF7-miR-18a-mimic with and without C59 or MCF7-negative control cells were counted and harvested after 4 days to record proliferation rates and to assay for TAP-1 protein levels.

### 2.10. Breast Cancer Cohort and Specimens Used for Analysis

Tumor samples from surgically excised breast tumors were selected from a non-consecutive case series from the Kidwai Memorial Institute of Oncology (KMIO), a regional cancer centre, wherein tumor blocks of 280 patients from 1982 breast cancer specimens examined at the department of pathology between 2010 and 2012 were collected from the archives. This is a retrospective study, and information was obtained from the pathological records that did not have all the information pertaining to clinical staging or follow up. The details of the clinicopathological characteristics and sequential exclusion of the tumor samples from the case series for various analyses have been described earlier [16]. Informed consent for use of the material for research was obtained from all patients, and the study was approved by the Institutional Ethics Review Board (IERB) at KMIO (IEC Reg. No.: S475/79-80). ER+ HER2- tumors from this case series were used for IHC analysis.

### 2.11. Immunohistochemistry (IHC)

The primary antibodies used for IHC are listed in Supplementary Table S2. IHC was performed as described previously [16]. Briefly, the sections were incubated with primary antibody for 1 h at room temperature. Sections were further incubated with secondary antibody (DAKO REALTM EnVisionTM) for 20 min as per the kit instructions, followed by development of the color using DAB (DAKO REALTM EnVisionTM) for 10 min. Appropriate positive and negative controls were run for each batch. Staining patterns of MMP9, CD68, CD4, CD8, and CD206 were evaluated by pathologists (J.S.P. and S.P.). Immunoreactivity of more than 1% of cells was considered the positive expression for the immune markers.

### 2.12. Statistical Analysis

Descriptive statistics were used for all clinical variables. The difference in gene expression levels was evaluated by the Mann–Whitney U test/Kruskal–Wallis test or the two-tailed Student’s *t*-test. Correlations were evaluated by Pearsons’s rank test. For in vitro graphical representations, the results are depicted as mean ± standard error of mean or standard deviation calculated from two or more experiments. Statistical analysis was performed by the Student’s *t*-test. For all tests, *p* < 0.05 was considered to be statistically significant. All statistical analysis was carried out using the software XLSTAT 2021.5 (Windows, accessed on 14 January 2021).

## 3. Results

### 3.1. Over-Expression of miR-18a Drives Immunosuppression in ER-Positive Breast Cancer Cells

**Figure 1:**
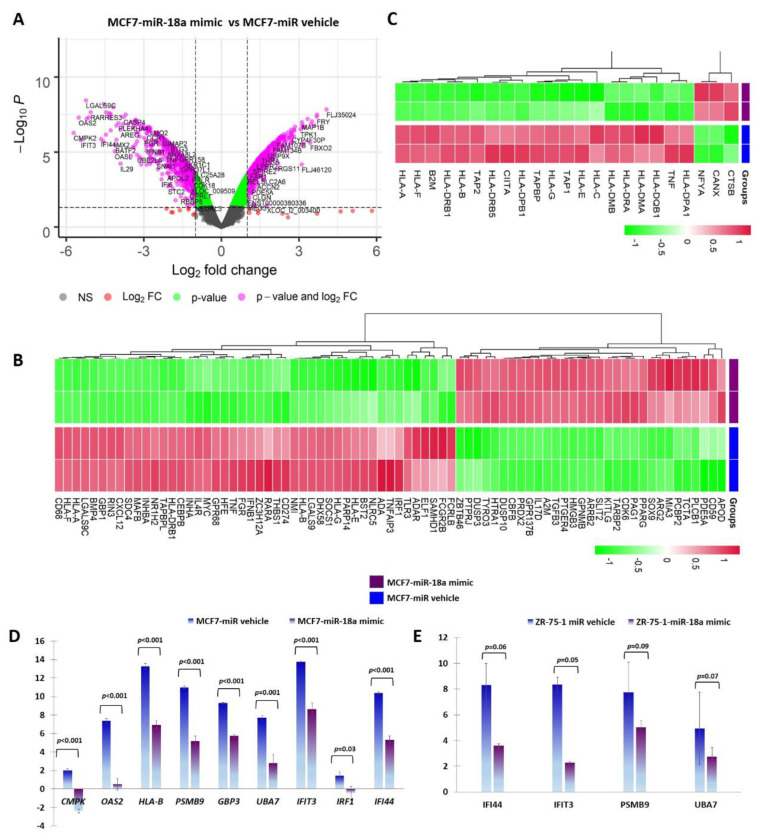
*miR-18a* over-expression mediates immune suppression in MCF7 and ZR-75-1. (**A**) Differentially expressed genes in MCF7-miR-18a-mimic vs. MCF7-negative control as obtained from microarray analysis. (**B**) Heat map demonstrating expression of immune-related gene signature in MCF7-miR-18a-mimic vs. MCF7-miR vehicle. (**C**) Heat map demonstrating expression pattern of genes regulating antigen presentation in MCF7-miR-18a-mimic vs. MCF7-miR vehicle. (**D**) q-PCR validation of down-regulation of representative immune-related genes in MCF7-miR-18a-mimic compared to MCF7-negative control. (**E**) q-PCR validation of down-regulation of representative immune-related genes in ZR-75-1-miR-18a-mimic compared to ZR-75-1-negative control. Values are mean ± S.E.M. (*n* = 3). Statistical analysis was performed by the Student’s t-test compared with the scrambled negative control.

miR-18a was over-expressed using a synthetic mimic for hsa-miR-18a-5p in ER+ cell lines MCF7 and ZR-75-1 as previously described [5]. To identify putative target genes and specific pathways regulated by over-expression of miR-18a, mRNA microarray analysis of RNA isolated from MCF7-miR-18a-mimic was performed. The effectiveness and specificity of miR-18a over-expression has also been described in detail previously [5]. Functional enrichment and pathway analysis suggested a global suppression of immune-related pathways (Figure 1A, B). Analysis of specific immune-related pathways suggested repression of antigen presentation (Figure 1C), cytokine production and signaling (Supplementary Figure S1), type I interferon signaling pathway (Supplementary Figure S2), and TAP1 binding and neutrophil activation (Table 1). Down-regulation of specific genes belonging to various immune pathways were also validated using q-PCR after miR-18a over-expression in MCF7 and ZR-75-1. The decreased expression of the following down-regulated genes associated with immune modulation were validated: *CMPK2* (*p* < 0.001-MCF7) and *IFI44* (*p* < 0.001-MCF7, *p* = 0.06-ZR-75-1); cytokine-signaling-associated genes *OAS2* (*p* < 0.001-MCF7), *PSMB9* (*p* < 0.001-MCF7, *p* = 0.09-ZR-75-1), and *GBP3* (*p* < 0.001-MCF7); interferon-signaling-associated genes *UBA7* (*p =* 0.001-MCF7, *p* = 0.07-ZR-75-1)*, IFIT3* (*p* < 0.001-MCF7, *p* = 0.05-ZR-75-1), and *IRF1* (*p* = 0.03-MCF7); and antigen-presentation-associated gene *HLA-B* (*p* < 0.001-MCF7) (Figure 1D,E).

### 3.2. ER-Positive Breast Tumors with High Levels of miR-18a Are Associated with an Immunosuppressed Stroma

**Figure 2:**
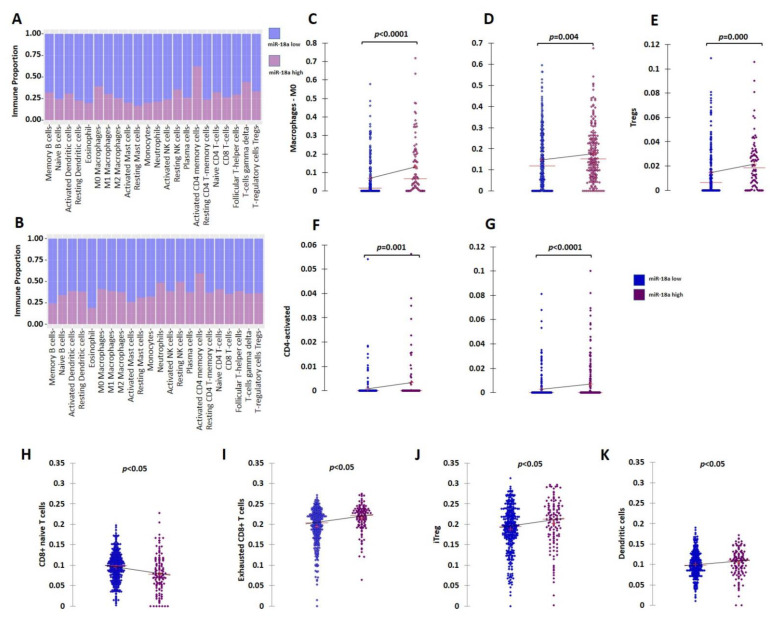
Analysis of immune cell proportions in ER+ miR-18a high- vs. low-expressing tumors. (**A**) Proportion plot after CIBERSORT analysis depicting immune cell proportions in ER+ tumors of the TCGA cohort (*n* = 333) segregated based on upper and lower quartiles of miR-18a expression. (**B**) Proportion plot after CIBERSORT analysis depicting immune cell proportions in ER+ tumors of the METABRIC cohort (*n* = 506) segregated based on upper and lower quartiles of miR-18a expression. (**C**) Scattergrams depicting associations between proportions of M0 macrophages and ER+ tumor groups of TCGA and (**D**) METABRIC segregated based on upper and lower quartiles of miR-18a expression as analyzed by CIBERSORT. (**E**) Scattergrams depicting associations between proportions of Tregs and ER+ tumor groups of TCGA. (**F**) Scattergrams depicting associations between proportions of CD4 activated cells and ER+ tumor groups of TCGA and (**G**) METABRIC. (**H**–**K**) Scattergrams depicting associations between immune cell type proportions and ER+ tumor groups of TCGA segregated based on upper and lower quartiles of miR-18a expression as analyzed by ImmuneCellAI. Statistical analysis was performed by Mann–Whitney U test. *p* < 0.05 is considered significant.

Further clinical validation for the immunosuppressive tumor microenvironment (TME) associated with ER+ tumors with high miR-18a levels was performed using external datasets of the TCGA and the METABRIC cohorts. The ER+ HER2- tumors of the TCGA (*n* = 479) and METABRIC cohorts (*n* = 883) were segregated based on the upper and lower quartiles of miR-18a expression. Immune cell identification was performed using CIBERSORT analysis, a deconvolution method that describes the cell composition of complex tissue from their gene expression profiles in tumors. The analysis in both the cohorts (TCGA; *n* = 333 and METABRIC; *n* = 506 emerged significant with *p* < 0.05) revealed that ER+ tumors with high miR-18a correlated with increased proportions of M0 macrophages (TCGA-*p* < 0.0001; METABRIC-*p* = 0.004), activated CD4 cells (TCGA-*p* = 0.001; METABRIC-*p* < 0.0001), and lesser memory B cells (METABRIC-*p* = 0.016) and monocytes (TCGA-*p* = 0.017). In the TCGA cohort, the miR-18a high tumors also correlated with a higher proportion of T-regulatory cells (*p* = 0.0002), a specialized subset of T cells that act to suppress immunity. The CD4/CD8 ratio was also observed to be higher in the tumors that expressed higher miR-18a levels in both the cohorts (TCGA-*p* = 0.001; METABRIC-*p* = 0.0002) (Figure 2A–G). These observations were further confirmed using ImmuCellAI, a gene-expression-based method for precisely estimating the abundance of immune cells with superior accuracy to other methods, especially the multiple types of T-cell subsets, in the TCGA cohort. The CD4/CD8 ratio was observed to be higher in miR-18a high tumors (*p* = 0.009) (Figure 2H) accompanied by an increased proportion of exhausted CD8 + T cells (*p* < 0.0001) (Figure 2I), induced Treg (iTreg) population (*p* = 0.01) (Figure 2J), dendritic cell population (*p* = 0.03) (Figure 2K), and decreased proportions of anti-tumor response eliciting Th17 cells (*p* < 0.0001). Thus, a significant overlap in the abundance of immune subsets was observed in the results generated by both the methods suggestive of an immune-suppressive pro-tumor microenvironment in ER+ tumors with high miR-18a levels.

### 3.3. MCF7 Cells with High miR-18a Levels Induce Pro-Tumorigenic M2 Phenotypic Differentiation and Possess Decreased Antigen Presentation Abilities That Are Reversed upon Wnt Inhibition

**Figure 3:**
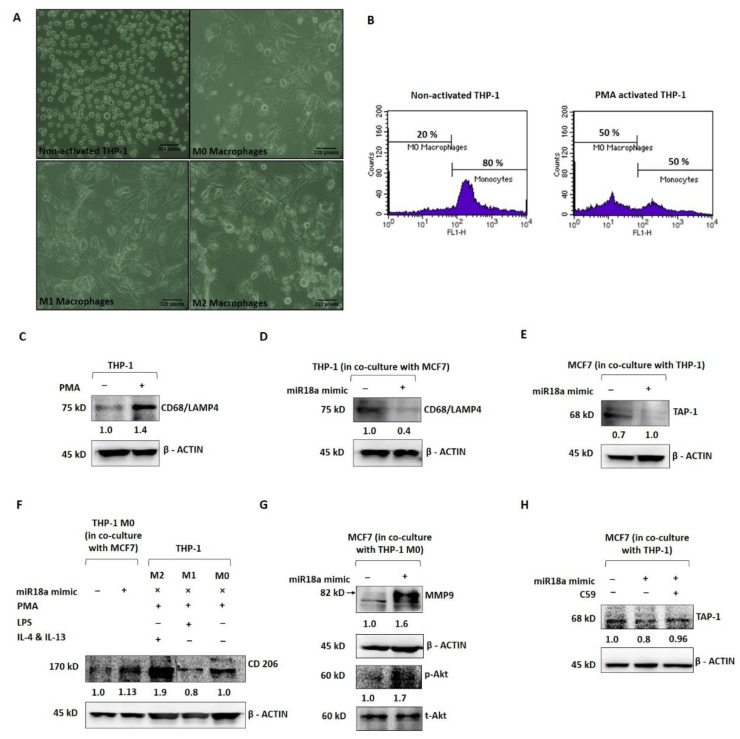
miR-18a over-expression mediates immune suppression in monocytic cell line THP-1 and promotes invasive and survival ability in ER+ breast cancer cells. (**A**) Morphological changes to THP-1 on PMA induction and morphological changes to M0 macrophages on treatment with LPS, IL-4, and IL-13, respectively, to facilitate differentiation to M1 and M2 macrophages, respectively. (**B**) Immunophenotyping by flow cytometry to assess level of expression of CD14 in THP-1 with and without PMA induction. (**C**) Expression levels of CD68 in THP-1 with and without PMA induction. (**D**) Expression levels of CD68 in THP-1 cells in co-culture with MCF7-miR-vehicle and MCF7-miR-18a-mimic. (**E**) Expression levels of TAP-1 in MCF7-miR-vehicle and MCF7-miR-18a-mimic co-cultured with THP-1. (**F**) Expression levels of CD206 in MCF7-miR-vehicle and MCF7-miR-18a-mimic co-cultured with THP1, control M2, M1, and M0 macrophages. (**G**) Expression levels of MMP9, p-Akt, and total-Akt in MCF7-miR-vehicle and MCF7-miR-18a-mimic co-cultured with THP1. (**H**) Expression levels of TAP-1 in MCF7-miR-vehicle, MCF7-miR-18a-mimic and MCF7-miR-18a-mimic-C59 inhibited, co-cultured with THP1. Values are mean ± S.E.M. (*n* = 3). Statistical analysis was performed by the Student’s *t*-test compared with the mimic negative control.

In order to mechanistically probe the tumor-promoting and immune-suppressive functions of high miR-18a in ER+ breast cancer, miR-18a was over-expressed in MCF7 and co-cultured with the THP-1 monocytic cell line to assess the immune-stimulating ability of miR-18a. As a positive control, THP-1 cells were induced to differentiate into the M0 phenotype using PMA as described in the methodology (Figure 3A). THP-1 after PMA induction expressed decreased levels of the monocyte marker CD14 by 30% (*p* = 0.1), as determined by immunophenotyping (Figure 3B) and increased levels of the macrophage marker CD68/macrosialin, which is a glycoprotein abundantly expressed by macrophages by 40% (*p* = 0.05) (Figure 3C). Further, the THP-1 cells co-cultured with MCF7-miR-18a-mimic and MCF7-miR-vehicle were probed for the expression of CD68, and it was observed to be 57% less in THP-1 cells co-cultured with MCF7-miR-18a-mimic compared to THP-1 cells co-cultured with MCF7-miR-vehicle (Figure 3D). On co-culture, it was also observed that transporter associated with antigen processing (TAP-1) decreased in miR-18a over-expressed MCF7 cells by 29% (*p* = 0.05) (Figure 3E). TAP-1 is critical for the major histocompatibility complex (MHC-I) antigen processing pathway and is associated with host-tumor surveillance levels. This is an indication of decreased immune stimulation of THP-1 monocytes that results in less differentiation to the M0 macrophage phenotype.

Further, PMA-induced and -differentiated THP-1 cells were co-cultured with MCF7-miR-18a-mimic and MCF7-miR-vehicle to assess the phenotypic changes elicited on these macrophages and transformation to the M1 or M2 phenotype. As a positive control, PMA-induced THP-1 cells were converted to M1 and M2 macrophages as described earlier (Figure 3A). CD206, a mannose receptor abundantly expressed by the M2 macrophages, was expressed up to 88% (*p* = 0.05) after induction using IL-4 and IL-13 (M2) when compared to LPS induction (M1), where CD206 expression decreased to 20% (*p* = 0.0009) (Figure 3F). The M2 macrophages also expressed more of the M2 gene *EGR2* (by 2-fold-*p* = 0.1) and less of the M1 gene *GPR18* (by 3-fold-*p* = 0.1) (Supplementary Figure S3). The PMA-induced THP-1 cells co-cultured with MCF7-miR-18a-mimic and MCF7-miR-vehicle were then harvested to assay for the expression of CD206. CD206 expression increased in PMA-induced THP-1 cells co-cultured with MCF7-miR-18a-mimic by 13% (*p* = 0.01), suggestive of the ability of high miR-18a-expressing cells to induce differentiation of M0 macrophages into the M2 phenotype (Figure 3F). MCF7-miR-18a-mimic and MCF7-miR-vehicle cells co-cultured with PMA-induced THP-1 (M0 macrophages) were also independently harvested to assay for proteins that correlate with invasiveness. The results showed a 64% increase in MMP9 levels (*p* = 0.05) in MCF7-miR-18a-mimic cells co-cultured with M0 macrophages (Figure 3G). On microscopic examination, MCF7-miR-18a-mimic cells appeared more viable and proliferative. This intrigued us to probe for activation of the PI3K-Akt survival pathway in these cells. *p*-Akt levels increased in the MCF7-miR-18a-mimic cells by 70% (*p* = 0.02) when compared to the MCF7-miR-vehicle-transfected cells co-cultured with M0 macrophages (Figure 3G). The results are suggestive of the existence of tumor–immune cell interactions, wherein high miR-18a-expressing breast cancer cells activate pathways that bring about selective immune-suppressed differentiation of immune cells that in turn increase the survival and invasiveness of breast cancer cells.

We have previously reported that miR-18a mediates the activation of the planar cell polarity (PCP) pathway, a branch of the Wnt signaling pathway that leads to increased activation of the JNK pathway and an eventual change in actin dynamics [5]. The Wnt pathway is also critical to immunomodulation. Multiple reports point towards the involvement of the Wnt pathway in shaping the immune cells and rendering a tumor-promoting microenvironment [17,18]. To test out the involvement of the activated Wnt pathway in these cells with over-expressed miR-18a, we used C59, a Wnt antagonist that inhibits porcupine (PORCN) required for Wnt palmitoylation, secretion, and biological activity. Upon Wnt pathway inhibition after miR-18a over-expression, we observed a significant decrease in cell viability and proliferation up to 49% (*p* = 0.005) (Supplementary Figure S4) in miR-18a over-expressed cells, which are otherwise highly proliferative as reported previously [5]. As described above, on co-culture of THP-1 cells with MCF7-miR-18a-mimic and MCF7-miR-vehicle, TAP-1 protein required for antigen presentation and processing significantly reduced in MCF7-miR-18a-mimic. However, inhibition of the Wnt pathway using C59 Wnt antagonist in MCF7-miR-18a-mimic led to a reversed effect with an increase in TAP1 expression up to 14% (*p* = 0.05), and TAP-1 levels became comparable to the MCF7-miR-vehicle-transfected cells (Figure 3H). This confirms the role of Wnt pathway activation mediated by high miR-18a levels in driving immunosuppression in ER+ breast cancer cells.

### 3.4. ER-Positive Tumors with High miR-18a Express High Levels of MMP9 and Expresses Markers Suggestive of a Tumor-Promoting, Immune-Suppressive Stroma

**Figure 4:**
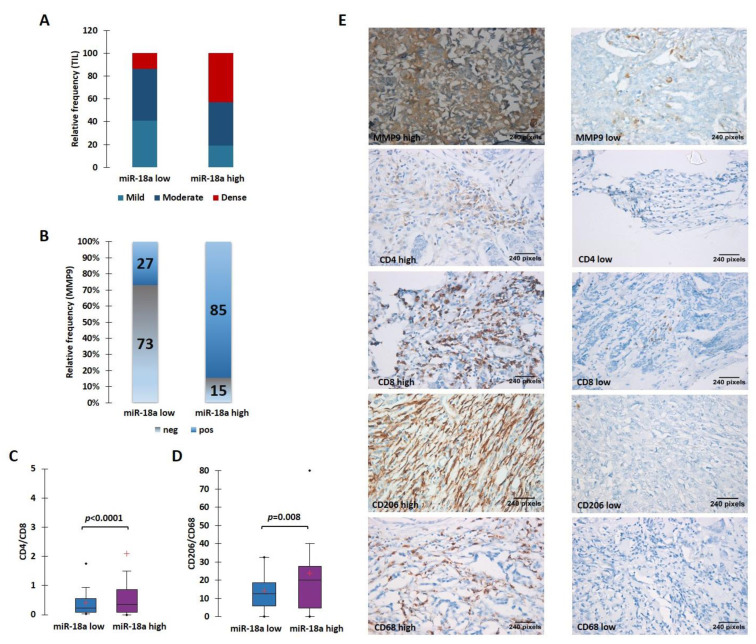
High levels of miR-18a in ER-positive tumor specimens correlate with a protein expression suggestive of an immunosuppressive ECM. (**A**) TIL density in ER+ miR-18a low and high tumors. (**B**) Expression levels of MMP9 in miR-18a low and high expressing tumors. (**C**) Association of CD4/CD8 in ER+ tumor groups segregated based on upper and lower quartiles of miR-18a expression. (**D**) Association of CD206/CD68 in ER+ tumor groups segregated based on upper and lower quartiles of miR-18a. (**E**) Representative images of MMP9, CD4, CD8, CD206, and CD68 stained sections. Statistical analysis was performed using two-sample comparison of variance test. *p* < 0.05 is considered significant. ‘+’ indicates mean and ‘∙’ indicates outlier.

The ER+ tumors from our case series were morphologically analyzed for the TIL density. The stromal immune infiltrate density was qualitatively scored as mild, moderate, and dense. To maximize the specificity, we chose to take a cut-off for miR-18a transcript at the third quartile (third quartile at 9.5) and divided the samples into miR-18a high (*n* = 22) and low (*n* = 59). We observed that miR-18a high tumors had the presence of denser TILs when compared to the miR-18a low tumors (*p* < 0.0001) (Figure 4A). These ER+ tumors (*n* = 54) were further examined for MMP9 expression by immunohistochemistry assays. In miR-18a high samples, 11/13 samples (85%) expressed high levels of MMP9 as opposed to 11/41 (27%) of miR-18a low tumor specimens (*p* < 0.0001) (Figure 4B). The miR-18a high and low tumor specimens (*n* = 15 each) were further used for IHC labeling with CD4 and CD8. We observed that the CD4/CD8 ratio was higher in the ER+ miR-18a high tumor specimens (*p* < 0.0001) (Figure 4C). We also used CD68, a pan macrophage marker, and CD206, a tumor-promoting M2 macrophage marker for IHC labeling. It was seen that the miR-18a high tumors expressed increased levels of CD206 and a higher CD206/CD68 ratio when compared to the miR-18a low tumors (*p* = 0.008) (Figure 4D,E), which further supports the in vitro findings. They also reflect the observations seen in tumors from TCGA and the METABRIC cohort using CIBERSORT and ImmuCellAI algorithms. The results are suggestive of miR-18a high tumors being associated with a dense but pro-tumorigenic immune-suppressed stroma.

## 4. Discussion

The role of microRNAs in mediating therapy resistance and metastasis during tumor progression is now well established [4,19]. miR-18a belongs to the miR-17-92 polycistron, which is one of the most potent oncogenic miRNAs. It is known to be highly expressed in multiple cancer types, including basal-like breast cancer [20,21]. We have previously reported that miR-18a is highly expressed by a group of ER+ tumors that have low ER protein expression [5]. These tumors have a poorer prognosis when compared to the miR-18a low tumors, which was validated in a case series of our tumors and the METABRIC cohort. Further, we were also able to establish mechanistically that miR-18a activates the planar cell polarity branch of the Wnt pathway that increases migratory ability through actin re-modeling using cell line model systems [5]. In this report, we further explored the de-regulation and immune suppression mediated by the high levels of miR-18a in these tumors.

The initial analysis was performed in miR-18a over-expressed breast cancer cell lines-MCF-7 and ZR-75-1. Microarray analysis indicated a global suppression of immune-related pathways, specifically antigen presentation, cytokine, and interferon-mediated signaling. These observations were further validated using tumor samples from external datasets of TCGA and METABRIC using CIBERSORT analysis. This analysis suggested that the ER+ breast tumors with high miR-18a levels have a higher CD4/CD8 ratio, higher proportion of T-regulatory cells, and exhausted CD8 + T cells. An elevated CD4/CD8 was found to be associated with tumor progression and poor survival in breast cancer patients. T-cell exhaustion is known to drive tumor progression and immune suppression in cancer; moreover, Tregs infiltration is also associated with shorter disease-free survival in breast cancer [22,23,24]. It is interesting to note that the ER+ miR-18a high tumors are enriched for the Luminal B tumors in both TCGA and METABRIC cohorts when compared to the miR-18a low tumors. This observation correlates with the finding reported earlier that miR-18a over-expression in ER+ cell lines drives proliferation [5]. However, further examination suggested that although the CD4/CD8 ratio is higher in the miR-18a high expressing Luminal B tumors, Tregs are abundantly present in the Luminal A tumors. This is an indication that these observations pertaining to the immunosuppression observed in miR-18a high tumors may not be only emerging from the predominant Luminal B tumors (Supplementary Tables S3–S5).

Additionally, the findings were also validated through IHC in a set of breast tumor specimens from our case series. We observed that the majority of the ER+ high miR-18a-expressing samples expressed MMP9, and these tumor specimens also expressed a higher CD4/CD8 and a higher CD206/CD68. The in vitro co-culture experiments further lend support to this hypothesis where miR-18a over-expressed cells were co-cultured with THP-1 cells to examine differentiation changes to THP-1 induced by high miR-18a levels. miR-18a over-expressed MCF7 showed less TAP-1 expression, which is required for antigen presentation. Moreover, THP-1 co-cultured with MCF7-negative control expressed more CD68, a macrophage marker suggesting that high miR-18a-expressing cells failed to stimulate THP-1 monocytes to macrophages, an indication that they are immunosuppressive. Further experimentation showed that the macrophages co-cultured with MCF7-miR-18a-mimic expressed more of the pro-tumorigenic M2 macrophage marker CD206, and co-culture-derived MCF7-miR-18a-mimic cells expressed more of the invasive marker MMP9 and activated PI3K-Akt pathway that aids in survival. This is an exemplary simulation of the in vivo TME where the miR-18a over-expressing ER+ tumor cells remain less immune-stimulating and inert, thereby enriching an immune milieu that is tumor promoting. This inciting niche in turn helps the tumor cells to survive, proliferate, and become more invasive, which may ultimately result in relapse and distant metastasis. The association of miR-18a and the AKT/mTOR signaling pathway in rendering increased proliferation and migratory abilities to breast cancer cells was recently described and hence lends support to our hypothesis [25]. We have thus been able to demonstrate the involvement of miR-18a at various levels of immune evasion mechanisms, including alterations in antigen presentation, dysfunction of effector cells, and subsequent changes to tumor cells.

Further, to confirm the role of the Wnt pathway in mediating these immunosuppressive effects, the Wnt pathway was blocked in miR-18a over-expressing cells, and co-culture experiments were performed. We observed that TAP-1 required for antigen presentation was restored by using Wnt-specific inhibitors to block the pathway. This suggests an involvement of the activated Wnt pathway in determining the quality of immune stroma in miR-18a over-expressing ER+ breast cancer. The role of Wnt in immune modulation and regulating the tumor-immune microenvironment is now well established [17,18]. We have previously demonstrated that miR-18a over-expression leads to decreased ER-based signaling and Wnt pathway activation. In addition, we have shown that ER repression alone may not trigger the activation of Wnt non-canonical signaling, but it’s suppression mediated by high levels of miR-18a is critical to activation of the Wnt pathway [5], implying that ER suppression and Wnt activation are two distinct but synergistic events that occur as a result of elevated levels of miR-18a. The Wnt pathway may be aberrantly regulated in cancer, but it is important to note that the activated Wnt pathway is also implicated in the maintenance of adult tissue homeostasis. Hence, there arise several safety concerns against the use of the Wnt pathway inhibitors for disease conditions [26]. Despite this, several Wnt-pathway-targeting therapeutic strategies are under development, and several of them are in the early phases of clinical trials [27,28]. Inhibition of Wnt could improve antigen presentation, CD8+ T-cell infiltration, and anti-tumor macrophage differentiation, which may yield a more promising scenario for employing immunotherapy in the ER+ breast cancer setting.

## Figures and Tables

**Table 1:** Down-regulated pathways in MCF7-miR-18a-mimic vs. MCF7-negative control.

Term ID	Term Name	P_adj_ (Query_1)
GO:0002376	Immune system process	2.366 × 10^−10^
GO:0001816	Cytokine production	1.008 × 10^−3^
GO:0060337	Type I interferon signaling pathway	3.148 × 10^−13^
GO:0046977	TAP binding	7.475 × 10^−5^
GO:0046978	TAP1 binding	4.037 × 10^−2^
GO:0034340	Response to type I interferon	3.039 × 10^−13^
GO:0060333	Interferon-gamma-mediated signaling pathway	9.448 × 10^−15^
GO:0034097	Response to cytokine	6.419 × 10^−14^
GO:0071357	Cellular response to type I interferon	3.148 × 10^−13^
GO:0034341	Response to interferon-gamma	2.400 × 10^−11^
REAC:R-HSA-91…	Interferon signaling	1.901 × 10^−14^
REAC:R-HSA-90…	Interferon alpha/beta signaling	3.665 × 10^−14^
REAC:R-HSA-87…	Interferon gamma signaling	1.166 × 10^−10^
REAC:R-HSA-12…	Cytokine signaling in immune system	7.598 × 10^−8^
REAC:R-HSA-16…	Immune system	2.061 × 10^−6^
REAC:R-HSA-98…	Antigen presentation: Folding, assembly, and pepti…	1.031 × 10^−4^
GO:0071346	Cellular response to interferon-gamma	1.570 × 10^−9^
GO:0002252	Immune effector process	2.439 × 10^−9^
GO:0002274	Myeloid leukocyte activation	1.286 × 10^−6^
GO:0045321	Leukocyte activation	2.703 × 10^−6^
KEGG:04612	Antigen processing and presentation	7.011 × 10^−4^
KEGG:04668	TNF signaling pathway	2.027 × 10^−2^

Table listing immune-system-related pathways down-regulated in MCF7-miR-18a-mimic vs. MCF7-negative control. Gene ontology and pathway analysis of DEGs were performed using ToppGene suite with the adjusted *p* ≤ 0.05 based on Benjamini–Hochberg false discovery rate.

## Data Availability

Not Available.

## Supplementary Materials

The following supporting information can be downloaded at: https://www.mdpi.com/article/10.3390/cells11101672/s1, Figure S1: Heat map depicting expression pattern of genes regulating cytokine signalling in MCF7-miR-18a-mimic and MCF7-miR-vehicle; Figure S2: Heat map depicting expression pattern of genes regulating interferon signalling in MCF7-miR-18a-mimic and MCF7-miR-vehicle; Figure S3: Gene expression of M1 (GPR18) and M2 macrophage marker (EGR2) in M0 macrophages transformed using IL-4 and IL-13 (M2) when compared to the M0 macrophages transformed using LPS (M1); Figure S4: Cell count recorded after a proliferation assay performed 72 hours after Wnt pathway inhibition post over-expression of miR-18a in MCF7; Table S1: List of primers used and their details; Table S2: List of antibodies used and their details; Table S3: Clinical details of ER+ tumor specimens from TCGA and METABRIC cohorts used for CIBERSORT Analysis; Table S4: Clinical details of ER+ tumor specimens from TCGA and METABRIC cohorts used for CIBERSORT analysis segregated based on miR-18a levels; Table S5: Results from CIBERSORT Analysis—segregated based on miR-18a and luminal subtypes.
