# Supplementary material for: miR-18a Mediates Immune Evasion in ER-Positive Breast Cancer through Wnt Signaling
Source: Cells. 2022 May 18;11(10):1672. doi: 10.3390/cells11101672 (PMC9139289; doi:10.3390/cells11101672)
Supplement: Supplementary file 1 [file cells-11-01672-s001.zip › cells-1671377-supplementary.pdf]

Supplementary Figures

Supplementary Figure S1: Heat map depicting expression pattern of genes regulating cytokine signalling in MCF7-miR-18a-mimic and MCF7-miR-vehicle

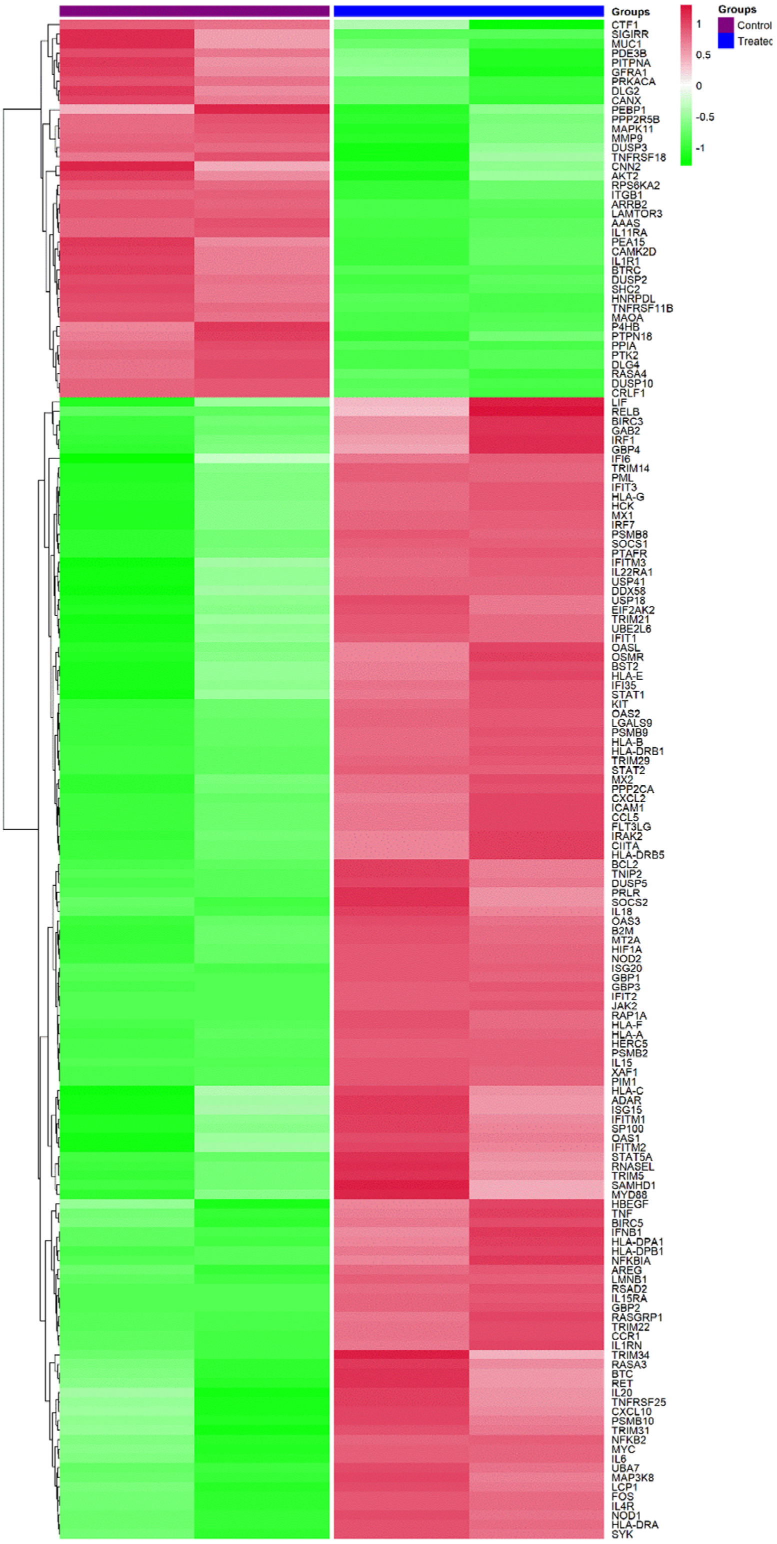

Supplementary Figure S2: Heat map depicting expression pattern of genes regulating interferon signalling in MCF7-miR-18a-mimic and MCF7-miR-vehicle

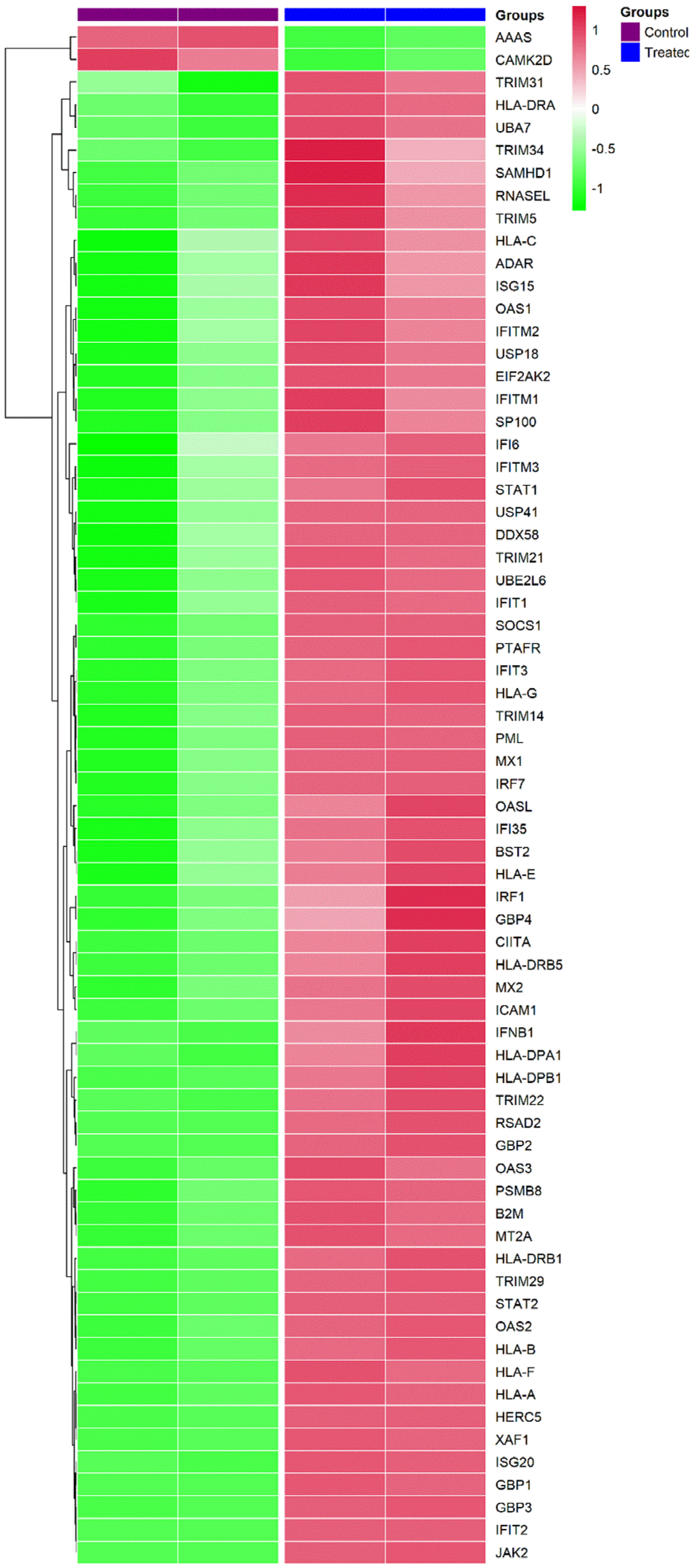

Supplementary Figure S3: Gene expression of M1 (*GPR18*) and M2 macrophage marker (*EGR2*) in M0 macrophages transformed using IL-4 and IL-13 (M2) when compared to the M0 macrophages transformed using LPS (M1)

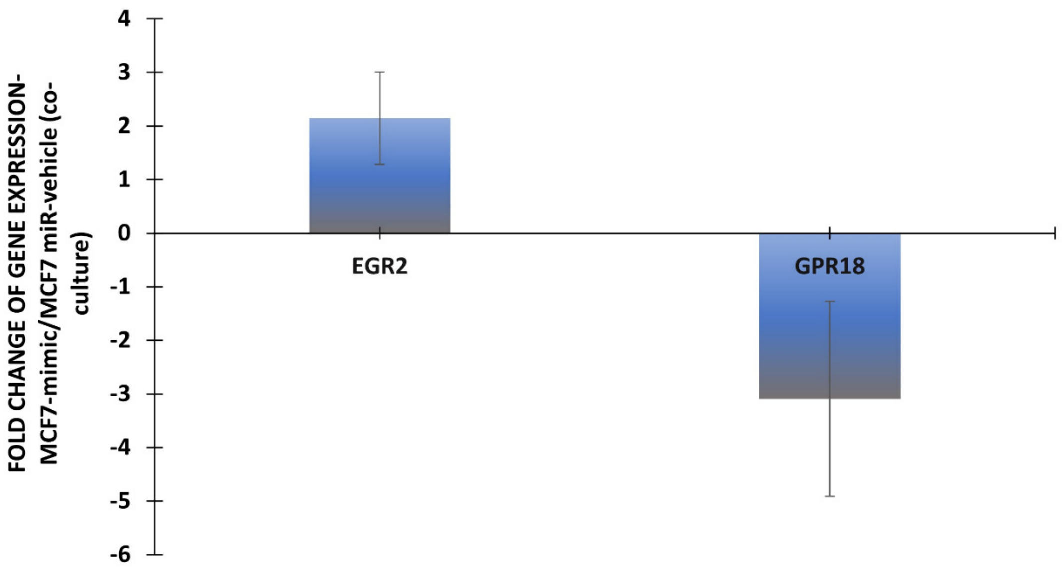

Supplementary Figure S4: Cell count recorded after a proliferation assay performed 72 hours after Wnt pathway inhibition post over-expression of miR-18a in MCF7.

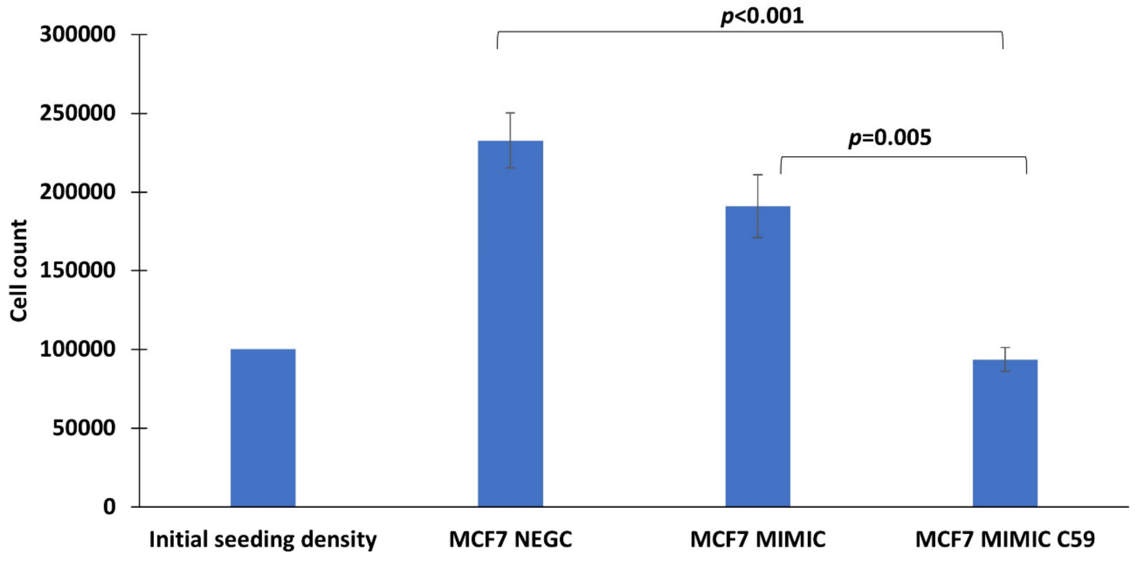

Supplementary Tables

Supplementary Table S1: List of primers used and their details

Sl no.	Gene	Primer Sequence
1	CMPK2 FP	5’-ccaggttgttgccatcgaag-3’
	CMPK2 RP	3’-cagccttaagtgaatctgccac-5’
2	OAS2 FP	5’-tgcaggaacccgaacagtt-3’
	OAS2 RP	3’-gtaccatcggagttgcctct-5’
3	HLA-B FP	5’-cagggctctgatgtgtctctcaca-3’
	HLA-B RP	3’-ggaggcgtgaagaaatcctgcatc-5’
4	PSMB9 FP	5’-gcacatctcatggtagctgg-3’
	PSMB9 RP	3’-tgtcgagtcagcattcctcc-5’
5	GBP3 FP	5’-agcactctcgtgtacaatagcatggg-3’
	GBP3 RP	3’-gatcggattcgatgtgtcagctctgt-5’
6	UBA7 FP	5’-atctagagctggcttcggcttctg-3’
	UBA7 RP	3’-acactccagacttccagggctttgt-5’
7	IFIT3 FP	5’-actccgatctcgctgagttc-3’
	IFIT3 RP	3’-agtagcgctgatgggattgt-5’
8	IRF1 FP	5’-aacttccaggtgtcacccat-3’
	IRF1 RP	3’-agcttcatgatgtcctcaggt-5’
9	IFI44 FP	5’-tgtaacgcatcaggctttggtggg-3’
	IFI44 RP	3’-ggcaggtatttgccatctttcccgt-5’

Supplementary Table S2: List of antibodies used and their details

Sl. No	Antibody	Product details	Dilution
1.	CD206/MRC1 (E2L9N) Rabbit mAb	91992S<br>Cell signalling technology	1:100
2.	CD14 (61D3) Mouse mAb (FITC Conjugate)	29943S<br>Cell signalling technology	1:25
3.	TAP1 Antibody	12341S<br>Cell signalling technology	1:1000
4.	p-Akt Rabbit mAb	2965S<br>Cell signalling technology	1:1000
5.	Akt (pan) Mouse mAb	2920S<br>Cell signalling technology	1:2000
6.	MMP9 Antibody	PA5-27191<br>Invitrogen	1:1000
7.	CD4-EP204 Rabbit mAb	PR013<br>PathnSitu Biotechnologies	Ready to use
8.	CD8-EP334 Rabbit mAb	PR223<br>PathnSitu Biotechnologies	Ready to use
9.	CD68 Mouse mAb	M0814<br>Agilent	Ready to use

Supplementary Table S3: Clinical details of ER+ tumor specimens from TCGA and METABRIC cohorts used for CIBERSORT Analysis

		**TCGA (%)**<br>**n=333**	**METABRIC (%)**<br>**n=506**
**PAM50 Classification**	Basal	3	4
	Her2	1	5
	LumA	71	45
	LumB	22	30
	Normal	3	15
**Menopausal status**	Pre	29	18
	Post	68	82
	Indeterminate	3	0

Supplementary Table S4: Clinical details of ER+ tumor specimens from TCGA and METABRIC cohorts used for CIBERSORT analysis segregated based on miR-18a levels.

		**TCGA** ***n*** **=333**		**METABRIC** ***n*** **=506**	
**PAM50 Classification**		miR18a low *n* =245	miR18a high *n* =88	miR18a low *n* =309	miR18a high *n* =197
	Basal	0	11	1	9
	Her2	1	1	4	6
	LumA	82	40	56	28
	LumB	15	45	20	46
	Normal	3	4	19	10
**Menopausal status**	Pre	27	35	14	24
	Post	70	63	86	76
	Indeterminate	3	2	0	0

Supplementary Table S5: Results from CIBERSORT Analysis- segregated based on miR-18a and luminal subtypes.

**Statistics**	**miR18a low Luminal A**	**miR18a low Luminal A**	**miR18a high Luminal B**	**miR18a high Luminal B**
**Number of observations TCGA**	196	35	34	38
**Median - Tregs**	0.005	0.008	**0.022**	**0.016**
**CD4/CD8**	0.009	0.023	0.015	**0.071**
**Number of observations METABRIC**	172	62	56	91
**CD4/CD8**	0.026	0.118	0.008	**0.345**

List of Acronyms used with their abbreviation

ER+ - Estrogen receptor positive
DEG - Differentially expressed genes
ICB - Immune checkpoint blockers
TNBC - Triple Negative Breast Cancer
mBC - Metastatic breast cancer
TILs - Tumor Infiltrating lymphocytes
ATCC - American Type Culture Collection
FC – Fold change
ImmuCellAI - Immune Cell Abundance Identifier
KMIO - Kidwai Memorial Institute of Oncology
IERB - Institutional Ethics Review Board
IHC – Immunohistochemistry
TME - Tumor microenvironment
MHC-I - Major Histocompatibility Complex
PCP - Planar cell polarity
PORCN – Porcupine
PMA - Phorbol-12-myristate 13-acetate
LPS – Lipopolysaccharide
ECM - Extracellular matrix

Details of reagents used in the study

Reagent	Company	Catalogue number
micrON hsa-miR-18a-5p mimic	Ribobio, China	miR10000072-1-5
micrON mimic NC #22	Ribobio, China	miR1N0000001-1-5
12-O-tetradecanoylphorbol-l3-acetate	Sigma-Aldrich	P1585
Lipopolysaccharides O111:B4	Sigma-Aldrich	L2630
Cell Culture Insert 0.4 µm	Corning	353494
Recombinant Human IL-13	Peprotech	200-13
Recombinant Human IL-4	Peprotech	200-04
C59, Wnt Antagonist	Abcam	ab142216
